# 3D Telomere Structure Analysis to Detect Genomic Instability and Cytogenetic Evolution in Myelodysplastic Syndromes

**DOI:** 10.3390/cells8040304

**Published:** 2019-04-02

**Authors:** Aline Rangel-Pozzo, Daiane Corrêa de Souza, Ana Teresa Schmid-Braz, Ana Paula de Azambuja, Thais Ferraz-Aguiar, Tamara Borgonovo, Sabine Mai

**Affiliations:** 1 Cell Biology, Research Institute of Oncology and Hematology, The Genomic Centre for Cancer Research and Diagnosis, University of Manitoba, CancerCare Manitoba, Winnipeg, MB R3E 0V9, Canada; aline.rangelpozzo@umanitoba.ca; 2 Arthur Siqueira Cavalcanti Hematology Institute (HEMORIO), Rio de Janeiro 20211-030, Brazil; dcsouzas@gmail.com (D.C.d.S.); tha_ferraz6@hotmail.com (T.F.-A.); 3 Hospital das Clínicas, Universidade Federal do Paraná, Curitiba, Paraná 80060-240, Brazil; ana.braz@hc.ufpr.br (A.T.S.-B.); apazamb@gmail.com (A.P.d.A.); tamaraborgonovo@yahoo.com.br (T.B.)

**Keywords:** myelodysplastic syndrome, cytogenetic evolution, genomic instability, 3D nuclear telomere organization

## Abstract

The disease course of myelodysplastic syndromes (MDS) features chromosome instability and clonal evolution, leading to the sequential acquisition of novel cytogenetic aberrations and the accumulation of these abnormalities in the bone marrow. Although clonal cytogenetic abnormalities can be detected by conventional cytogenetics in 50% of patients with MDS, such distinguishing patterns are lacking in the other 50%. Despite the increase in the prognostic value of some biomarkers, none of them is specific and able to discriminate between stable and unstable patients that subsequently progress to acute myeloid leukemia. This pilot study aimed to investigate the potential use of the 3D telomere profiling to detect genomic instability in MDS patients with or without clonal cytogenetic evolution. The comparison between different time points in patients with cytogenetic changes showed that in the CD34+ MDS cells, there was a significant decrease in the total number of telomeric signals, the average intensity of signals and the total intensity of telomeres. By contrast, the number of aggregates increased during cytogenetic evolution (*p* < 0.001). This pattern was observed only for MDS patients with cytogenetic evolution but was absent in patients without cytogenetic changes. In conclusion, we demonstrated that the 3D nuclear telomere organization was significantly altered during the MDS disease course, and may have contributed to cytogenetic clonal evolution.

## 1. Introduction

The pathogenesis of myelodysplastic syndrome(s) (MDS) is a multistep process characterized by the acquisition of genetic aberrations with increasing malignant potential of cells and risk of progression to acute myeloid leukemia (AML) [1]. The MDS disease course involves chromosome instability and clonal evolution that lead to the sequential acquisition of novel cytogenetic aberrations and the accumulation of these abnormalities in cells of the bone marrow [2]. Cytogenetic clonal evolution is defined by the presence of an abnormal clone in a patient whose karyotype was initially normal or additional aberrations in a patient with a karyotype that was already abnormal at the time of diagnosis [3]. This clonal evolution follows different and often disease-specific patterns during transformation to AML and relapse that are not completely understood, and is associated with a worse prognosis as compared to cases without cytogenetic changes [2]. Although clonal cytogenetic abnormalities are detectable in 50% of patients with MDS by conventional chromosome banding, the other 50% of patients lack such discriminatory patterns [4]. Despite the increase in the prognostic value of some biomarkers in predicting patient outcome and therapeutic responses to treatment in the last few years, none of these biomarkers are specific for MDS or are able to discriminate stable patients from unstable patients who subsequently progress to AML.

The 3D nuclear organization of telomeres allows for differentiation between normal and tumor cells; indeed, alterations in telomere architecture and telomeric dysfunction are hallmarks of chromosomal and genomic instability [5,6,7]. In previous work from our group, we showed that alterations in the 3D organization of telomeres are a common feature in cancer cells of different cancer types and that changes in 3D nuclear architecture of telomeres in tumor cells can be associated with the advancement and aggressiveness of such tumors at that particular time [8,9]. Therefore, we envisioned that 3D telomere profiling may provide a promising tool for better understanding of leukemogenesis and finding new therapeutic approaches for myelodysplastic syndrome [8]. The following blind pilot study aimed to investigate the potential use of 3D nuclear telomere organization in detecting genomic instability in MDS patients with or without clonal cytogenetic evolution.

## 2. Materials and Methods

### 2.1. Patient Samples

Bone marrow smears were obtained from a total of 15 patients diagnosed with MDS in the Hospital de Clínicas da Universidade Federal do Paraná (Paraná, PR, Brazil) and the Instituto Estadual de Hematologia Arthur de Siqueira Cavalcanti in Rio de Janeiro (Rio de Janeiro, RJ, Brazil), from 2003 to 2017, at diagnosis and during the course of the disease. We included samples of patients whose bone marrow cells were cytogenetically analyzed at least twice: once at the time of diagnosis and later during the course of the disease (all time points were before treatment). The time periods between the samples collection varied between 20 days and 3 months (Supplementary Table S1). We included patients with an abnormal karyotype as well as patients with a normal karyotype at the time of diagnosis. All the samples were collected during their clinical follow-ups with informed consent. No patients were previously diagnosed with other hematological diseases. Age, sex, WHO classification 2008, IPSS-R, and cytogenetics were recorded for each patient at diagnosis and during the disease course and AML transformation status. All patients retained the same WHO classification during all time points of cytogenetic analyses. The chromosomes were classified according to the International System for Human Genetic Nomenclature [10]. Informed consent was obtained from all the patients in accordance with the declaration of Helsinki. This study received approval by the Research Ethics Board on human studies in Brazil (CAE: 87629318.3.0000.0096 and 444/18). The median age of the patients was 53 years (range 5–82 years). All clinical information is presented in Table 1 and Supplementary Table S1.

### 2.2. Co-Immuno FISH and Imaging

The bone marrow smears slides were fixed with 3.7% formaldehyde/1 × PBS for 20 min, washed three times with 1 × PBS for 5 min, and blocked with 4% BSA in 4× saline-sodium citrate (SSC) for 15 min. The cells were incubated with rabbit anti-CD34 antibody (ab81289, ABCAM) as a primary antibody and Goat Anti-Rabbit IgG H&L-Alexa Fluor^®^ 488 (ab150077, ABCAM) as a secondary antibody. Three-dimensional quantitative fluorescence in situ hybridization was performed using Cy3- labeled peptide nucleic acid (PNA) probe (Dako, Glostrup, Denmark) as previously described [5]. The nuclei were counterstained with 4′,6-diamidino-2-phenylindole (DAPI) and coverslips mounted using VECTASHIELD (Vector Laboratories, Burlington, Ontario, Canada). We analyzed 30 CD34 positive interphase nuclei per time point using an AxioImager Z2 microscope (Carl Zeiss, Toronto, Ontario, Canada). A 63×/1.4 oil objective lens (Carl Zeiss Canada Ltd.) was used for image acquisition. Sixty z-stacks were acquired at a sampling distance of x,y: 102 nm and z: 200 nm for each slice of the stack. The ZEN 2.3 software (Carl Zeiss Canada Ltd.) was used for 3D image acquisition and processing (the constrained iterative algorithm was used for deconvolution). Deconvolved images were analyzed using the Teloview v1.03 software program [11] (3D Signatures Inc., Toronto, ON, Canada). TeloView (3D Signatures Inc.) loads 3D images and determines telomeric signal intensity (telomere length), the number of telomeric signals, the number of telomere aggregates, and *a/c* ratio [12].

TeloView™ (3D Signatures Inc.) calculates the center of gravity and the integrated intensity of each telomere, the latter determines the length of the telomere [12]. TeloView (3D Signatures Inc.) analyzes five parameters for each sample: (a) telomeric signal intensity (the relative fluorescent telomeric signal intensity represents the length of the telomeres in arbitrary units, since the intensity of the PNA probe hybridization signals is directly proportional to the length of the telomeric DNA); the average intensity illustrates the average length of telomere signals detected per nucleus per sample (30 nuclei/sample); (b) number of telomeric signals (total number of telomeres present in each cell); (c) number of telomere aggregates (TA) (clusters of telomeres found in close proximity to each other which cannot be further resolved as separate entities by TeloView (3D Signatures Inc.) as conventional microscopy has an optical resolution limit of 200 nm); (d) nuclear volume of each cell (measured by nuclear DAPI staining in the x, y and z dimensions); (e) *a*/*c* ratio, defining the nuclear space occupied by telomeres can be represented by three axes of length a, b, and c. The ratio between the a and c axes, the a/c ratio, is dynamic, and changes at different stages of the cell cycle (G_0_/G_1_, S, G_2_) [6,7,11,12,13,14].

### 2.3. Statistical Analyses

The telomeric parameters (number, length, telomere aggregates, nuclear volume, *a/c* ratio, etc.) were compared between different time points using a nested factorial analysis of variance followed by a least-square means multiple comparison. Graphical presentations indicated the *p*-value for the overall test of differences across 4 time points. Chi-square analysis compared the percentage of interphase telomeric signals intensities at defined quartile cut-offs. A significance level was set at 0.05. 

## 3. Results

### 3D Nuclear Architecture of Telomeres Has Changed During Cytogenetic Evolution

**Figure 1:**
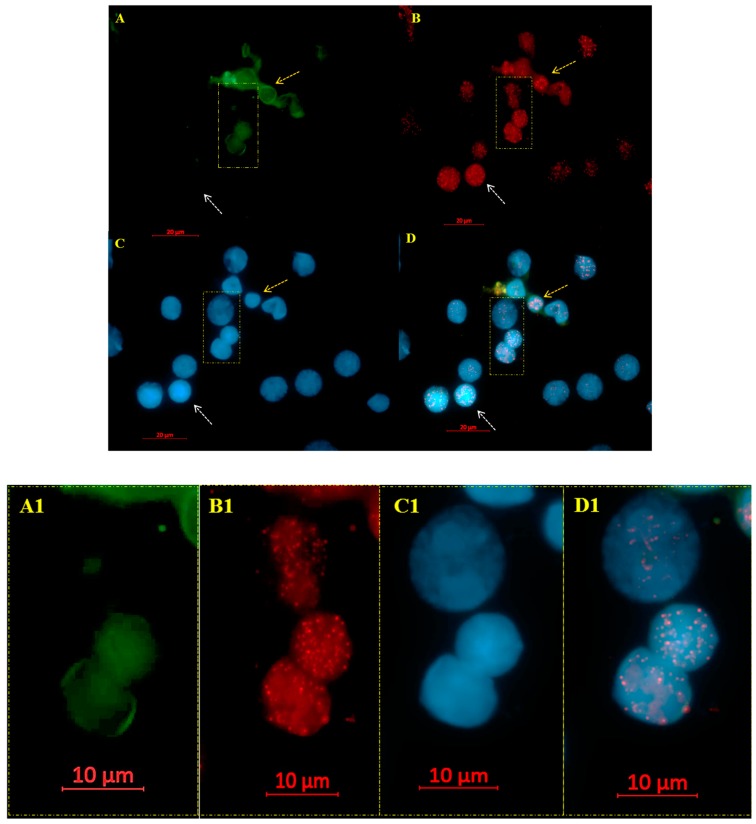
Telomere signals in CD34+ MDS cells (**A**–**D** with magnified view in **A1**–**D1**). (**A**) The MDS CD34 positive cells fluoresce green (see arrows in yellow), whereas the other hematopoietic cells remained unstained (see arrows in white). The telomeres, hybridized with Cy3-labeled PNA probes, appear as red signals (**B**). The nuclei are counterstained with DAPI (blue) (**C**). (**D**) Merge of FITC (CD34), Telomeres (CY3) and DAPI.

Genomic instability, disease progression, and relapse are commonly accompanied by cytogenetic changes that contribute to the development of subclones resistant to chemotherapy or even new clones [15]. To investigate the potential of 3D telomere profiles as a possible biomarker to quantify genomic instability and stratify MDS patients into subgroups [8], we analyzed fifteen MDS patient samples at diagnosis and during disease course, of which only ten had karyotyping changes or clonal evolution. The majority of the patients were adult males classified as intermediate-risk (Table 1). CD34+ antibody positivity was used to identify early hematopoietic cells, since the CD34+ cell population is enriched in hematopoietic stem cells [16]. MDS cells were differentiated from other hematopoietic cells based on the intensity of green fluorescence signals emitted by the CD34 antibody (Figure 1).

To examine the 3D nuclear telomere architecture in MDS cells, 3D Q-FISH was performed. After image acquisition and deconvolution, 30 nuclei for each time point were analyzed with TeloView (3D Signatures Inc.) [11] to determine the parameters reported in Material and Methods. The telomeres were visualized as red dots (Figure 1B,B1).

First, we evaluated the total number of telomere signals, which corresponds to the number of telomeres, in CD34+ MDS cells during the disease course. Cancer cells commonly exhibit both an altered number of telomeres per cell and a decrease in telomere length compared to normal cells [17]. We found that the total number of telomere signals decreased at the subsequent time points when compared with that at diagnosis for each patient with cytogenetic changes.

**Figure 2:**
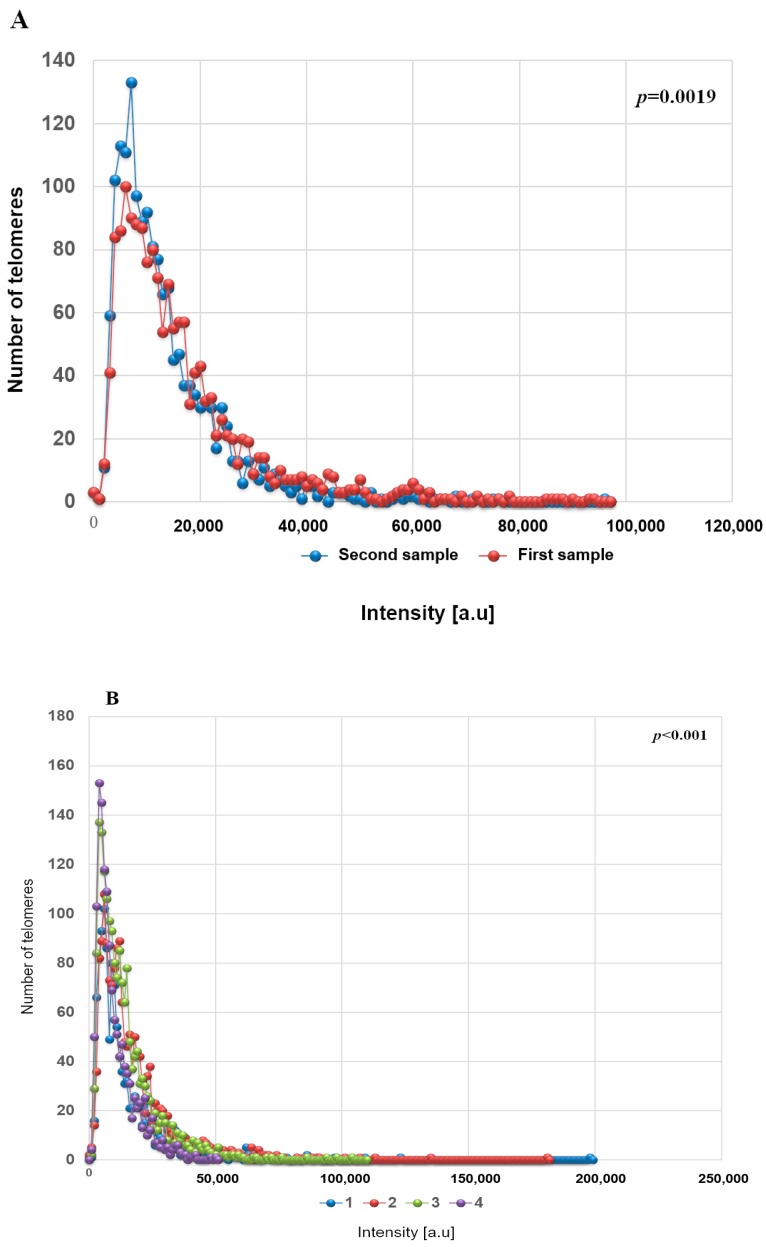
Telomere intensities change with disease progression (**A**,**B**). (**A**) First sample (red) obtained at time of diagnosis and second sample (blue) obtained during later follow-up show distinct profiles with respect to the telomere intensities and number of telomeres in the low-intensity range. Patient number 7. (**B**) First sample (1) obtained at time of diagnosis and next samples (2, 3 and 4) obtained during later follow-up after cytogenetic evolution. *p*-values included in each graph indicate the significance when comparing the first with the last sample. (first time point with last time point collected) Patient number 1. *p* values for all other comparisons are shown in Supplementary Table S2.

Next, we assessed the total intensity of telomere signals, proportional to telomere length, and noted the same significant differences between time points. The 3D telomere profile of CD34+ MDS shows that after acquisition of additional cytogenetic abnormalities, the telomeres become shorter, as indicated by an increase of telomere with low signal intensities (Figure 2 and Supplementary Table S2). In Figure 2, telomere length (signal intensity, x-axis) is plotted against the number of telomeres (y-axis) for all cells analyzed at each time point. Signals are grouped by their intensity level and this gives a picture of the telomere distribution in each sample or time point. For normal lymphocytes, for example, this plot usually has small peaks between 0 and 20,000 a.u (arbitrary units of relative fluorescence intensity), in which the number of telomeres per nucleus on the y-axis range between 5 and 25 [9]. For lymphocytes, most of the telomere signals have high relative intensities, with signals detected up to 120,000 a.u [9].

**Figure 3:**
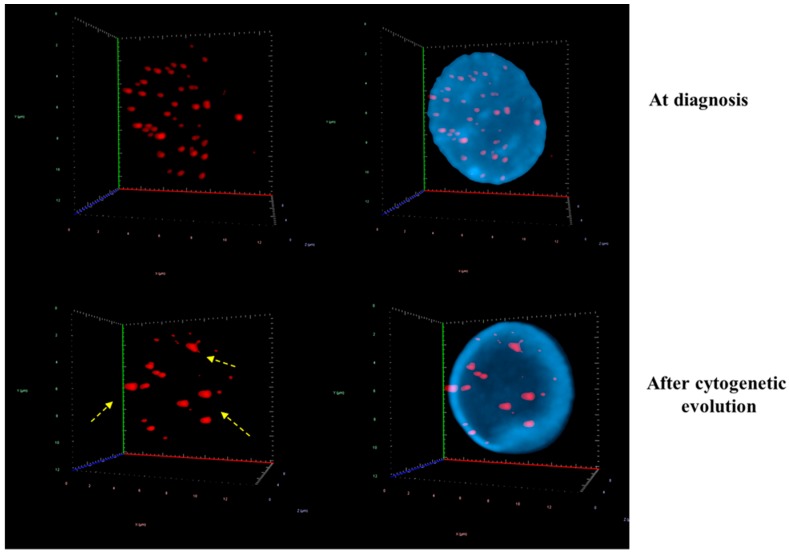
Evolution of 3D nuclear architecture during the MDS disease course. The left side shows representative 3D nuclear telomere distribution (red) within the counterstained nucleus (blue). During cytogenetic evolution, the 3D nuclear architecture undergoes distinct changes: The number of signals decrease and the number of telomere aggregates increase. The yellow dashed arrows indicate telomere aggregates (for details, see text and Supplementary Table S1 (Patient number 1).

**Figure 4:**
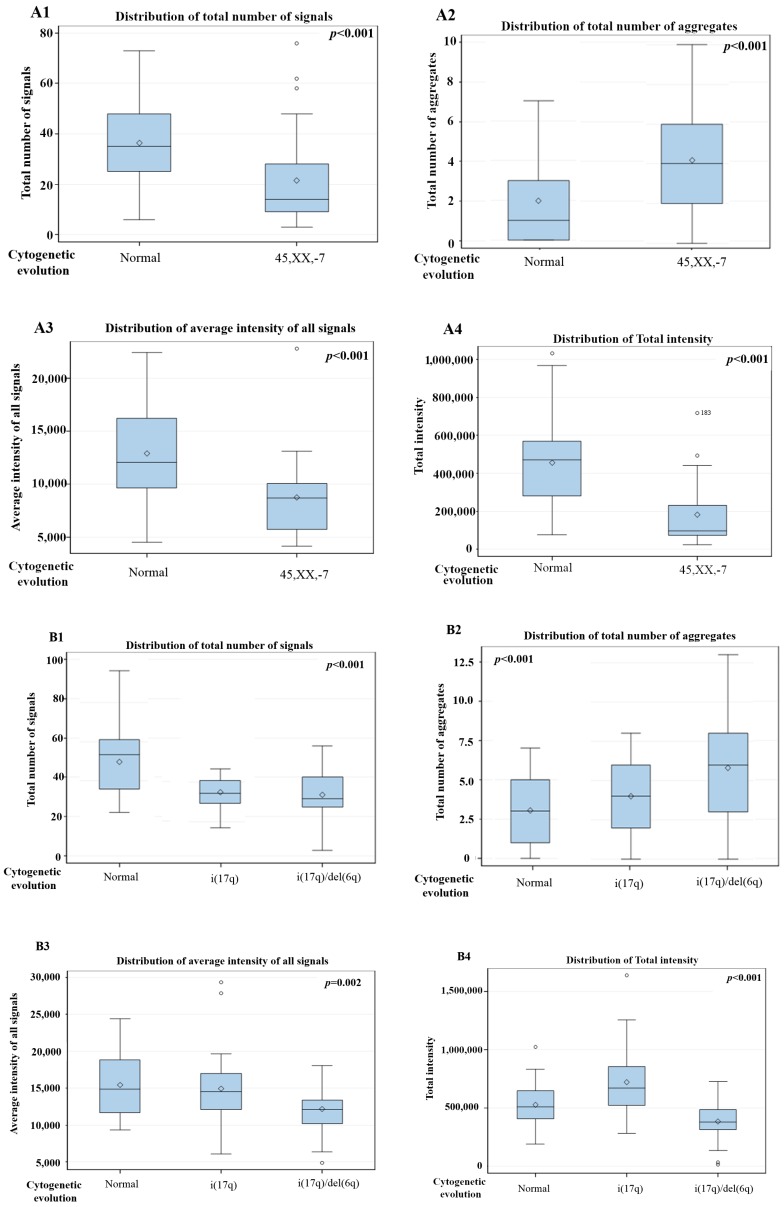

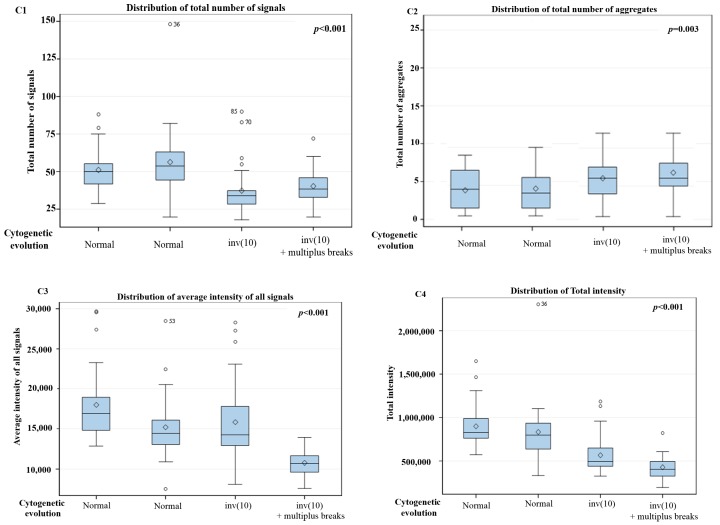
3D telomere profiling during cytogenetic evolution in MDS—two time points (**A1–A4**), three time points (**B1–B4**) and four time points (**C1–C4**) are shown after comparison of total number of signals, total number of aggregates, average intensity of signal and total intensity. *p*-values included in each graph indicate the significance when comparing the first sample with the last sample (first time point with last time point collected). *p* values for all other comparisons are shown in Supplementary Table S2. Patient number 2 (P2) is shown in (**A**), P4 in (**B**) and P1 in (**C**).

The comparison between different time points in 10 patients with cytogenetic changes demonstrated that the CD34+ MDS cells had a decreased in total number of signals, average intensity of signals and total intensity of telomeres. However, the number of telomeric aggregates, defined as clusters of fused telomeres or telomeres in close illegitimate proximity that cannot be further resolved as separate entities at an optical resolution limit of 200 nm, increased during cytogenetic evolution (Figure 3 and Figure 4). The p-values included in each graph represent the significance of the difference in telomere architecture (TeloView parameters) between the first and last time point. All other comparisons (and associated *p*-values) between time points are shown in Supplementary Table S2.

This pattern (↓ total number of signals, ↓ average intensity of signals, ↓ total intensity of telomeres and ↑ number of telomeric aggregates) was observed in all MDS patients with cytogenetic changes (10 patients in total). Telomeres in normal interphase nuclei do not overlap. Each telomere is found in a specific 3D space, and they do not form clusters or aggregates [7]. The TeloView program (3D Signatures Inc.) readily recognizes telomere aggregates, then calculates the average intensity of telomeres by analyzing the smaller telomeres, which represent the majority of telomeres. Smaller telomeres are interpreted as single copies while larger telomeres are interpreted as aggregated copies. Although the copy number can theoretically be calculated by dividing the integrated intensity of each telomere in a cluster by that of the specific telomere aggregate, aggregate formation usually decreases the total number of signals. Since aggregates are recognized as just one signal, they usually also decrease the total intensity of signals due to telomere sequence overlaps. 

Measurements of telomere positions in the 3D space are able to give a precise value to telomeres in different phase of the cell cycle [11]. The *a/c* ratio represents telomeric distribution in nuclei, with a low *a/c* ratio representing an oblate spheroid shape while a high *a/c* ratio corresponds to a disk-like shape. A high *a/c* ratio indicates that the cells are close to division (representing cells in G_2_ and G_2_/M), while small *a/c* ratios represent cells in G_0_/G_1_ and S [11,18]. The nuclear distribution of telomeres changes in G_2_ with the telomeres aligning in the center of the nucleus and forming a telomeric disk [18]. At this time, the *a/c* ratio is large, typically close to 14 ± 2 in normal lymphocytes [7]. Other TeloView (3D Signatures Inc.) parameters, such as nuclear volume and *a/c* ratio also changed for each patient at the subsequent time points. The volume of the cell nucleus can be measured from the image of DNA-based dyes. We observed a significant increase in the nuclear volume and *a/c* ratio for 8/10 patients with karyotype changes. In our study, the increase in the *a/c* ratio suggests that the cells are cycling or dividing more after each time point since more cells were found in the G_2_/M phase. This increase can be linked to the acquisition of cytogenetic abnormalities since short telomeres induce aggregates, which consist of fused telomeres or telomeres in very close illegitimate proximity.

**Figure 5:**
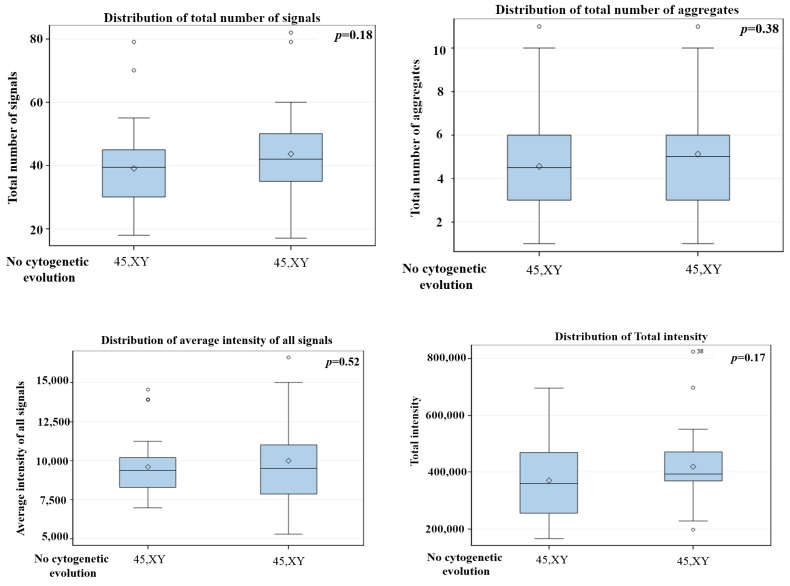
3D telomere profiling during disease course in MDS without cytogenetic changes—two time points are shown after comparison of total number of signals, total number of aggregates, average intensity of signals and total intensity. The *p* values are in each graph, total number of aggregates, average intensity of signal and total intensity. *p*-values included in each graph indicate the significance when comparing the first with the last sample. (first time point with last time point collected). *p*-values for all other comparisons are shown in Supplementary Table S2. Patient number 9 (P9).

Supplementary Table S2 summarizes all telomeres parameters for the fifteen MDS patients. We were able to distinguish two types of telomeric dysfunction in MDS cells, namely the shortening of telomeres (decrease of number of signals) and the formation of telomere aggregates. Both types of telomeric dysfunction can lead to bridge-breakage fusion cycles contributing to acquisition of additional chromosomes alteration [7]. Five MDS patients without cytogenetic changes during disease course showed no significant changes in telomere parameters between time points (*p* > 0.05) (Figure 5).

## 4. Discussion

To our knowledge, this pilot study is the first to apply 3D telomere analysis to follow the cytogenetic evolution in MDS. Cytogenetic abnormalities detected at primary diagnosis may change over time, evidently a frequent event in MDS patients [3,19,20,21]. Those changes also reflect the heterogeneous and dynamic nature of the disease [3,19,20,21]. Although cytogenetic evolution is a more common event in patients who had unfavorable cytogenetic at diagnosis as compared to patients with favorable or intermediate cytogenetic profiles [2], we did find a high frequency of cytogenetic evolution in MDS patients with an intermediate cytogenetic classification. The majority of clonal evolution events is observed during the first 12 months, which is comparable to the results published previously [22]. The altered karyotype at baseline appears to predispose toward the acquisition of further alterations, as seen in the work of Neukirchen et al. [22]. The cumulative 5-year incidence rate of clonal evolution was only 11.7% in patients presenting with a normal karyotype but was 27.8% in those presenting with any chromosomal aberration. The assumption that cytogenetic clonal evolution indicates a malignant clone with more pronounced chromosomal instability and more aggressive behavior, is supported by the higher risk of developing AML. However, Neukirchen et al. [22] detected that only 33% of the cases of clonal evolution had clinical progression to AML.

A number of previous studies aimed to characterize clonal evolution patterns and investigate their prognostic impact [23,24,25,26,27,28,29,30]. Tricot et al. [23] showed four clonal cytogenetic evolution patterns, which include stable clones with low proliferative activity, unstable clones with low proliferative capacities, stable clones with more pronounced proliferative capacities, and unstable clones with pronounced proliferative capacities. Schanz et al. [24] confirmed with a large MDS cohort that cytogenetic clonal evolution represents an independent adverse prognostic factor for patients with MDS and related myeloid disorders. In addition, their study emphasized the value of sequential cytogenetic analyses to monitor cytogenetic evolution in MDS patients during regular follow-ups [24].

The present study is the first to propose the use of the 3D telomere profiling to detect clonal stability and clonal evolution in MDS. A crucial advantage of 3D nuclear telomere profiling is the ability of the assay to use non-dividing cells and thus interphase nuclei. Our technology can provide important information about genomic instability considering that 15%–20% MDS bone marrow cultures lack metaphases suitable for cytogenetic analyses and half of patients with MDS have a normal karyotype [25]. Therefore, we aimed to only analyze CD34 positive cells in the bone marrow. Like AML, MDS is considered a clonal stem cell disorder. In AML, the stem cell has certain conserved features that are unique to the leukemic stem/progenitor cell but are not found in normal hematopoietic stem cells. Whether early MDS stem/progenitors share such unique features remains to be elucidated. Therefore, the current consensus is that CD34+ cells should be the cell type to be examined as the MDS “stem” cell.

All patients with cytogenetic evolution had significant differences in at least two of the five telomeres parameters when they showed disease progression. As presented for patient P1, the comparison between time points showed a progressive shortening of telomeres. Importantly, the comparison between the first two time points (patient P1) when the cytogenetic results were still normal karyotype already demonstrate a significant difference (*p* = 0.02), which might point to unstable disease and increased potential of cytogenetic evolution. The same was demonstrated for the patient (P4) with three time points. In our study, cytogenetic changes were associated with a decrease in both telomere numbers and telomere lengths. Our assumption is that the number of telomere decreases due to the process of telomere shortening and/or the formation of telomere aggregates. Although the decrease in the number of signals and the increase in telomere aggregates was a common feature between time points, for five patients without cytogenetic changes this difference was not significant.

There are few explanations why we couldn’t find the same telomere changes in MDS patients without cytogenetic evolution: (1) That those patients could in fact be classified as stable patients (as in the case of P12, P13, P14 and P15, for example) or (2) the levels of genomic instability were already high and thus did not change further between time points (as in the case of P9, for example). This point further underscores the importance of a possible future integration of 3D telomere profiles into already established risk stratification systems. Certainly, our cohort was small and such assessment requires validation in a larger cohort.

Nevertheless, when compared with existing studies, our pilot cohort was indeed a unique cohort as we were able to evaluate MDS patients with cytogenetic evolution before administration of any treatment. Although MDS progression is considered a step after acquisition of novel clonal aberrations [30,31,32,33], no correlation with AML transformation was investigated. While a larger study is necessary to assess the ability of this technology in early detection of the progression from MDS to AML, it was seen that 60% of the patients with clonal cytogenetic evolution in our cohort already progressed to AML and 30% died before progression (P3, P7, P8, P11) (Supplementary Table S1). The remaining patient, patient number 6, is still alive but was recently diagnosed and at present, no progression has been detected. 

The remodeling of 3D nuclear telomere architecture is one of the earliest events in telomere dysfunction in cancer cells and can occur at an early phase of the leukemogenesis [13]. In our current study, all patients with cytogenetic evolution showed significant changes in the telomere parameters, and we were able to observe a distinct shortening of telomeres and increase of telomere aggregates during the course of the MDS disease. The telomere aggregates can lead to further acquisition of chromosomal abnormalities such as deletions, gene amplification and non-reciprocal translocations [34]. Some of the aggregates fuse and these end-to-end fused chromosomes cannot appropriately segregate during cell division, leading to anaphase bridges and subsequent chromosome breakages. These breakage-bridge fusion cycles continue until no more free telomere ends are available [5,7]. Critically short telomeres have been implicated in the formation of telomere aggregates in Hodgkin’s lymphoma and other diseases [9,35,36].

The nuclear space occupied by telomeres can be represented by three axes of length *a*, *b*, and *c*. The *a*/*c* ratio was observed to increase between time points in our cohort. This result suggests that the cells are cycling or dividing more after each time point since more cells were found in the G_2_/M phase. The telomere length does not change during the cell cycle. However, the *a/c* ratio is dynamic, and changes at different stages of the cell cycle (G_0_/G_1_, S, G_2_). A previous study from our group had demonstrated the concordance between the *a*/*c* ratio and cell cycle phase [14]. The authors showed that the proliferation index measured by Ki-67 staining was confirmed by the a/c ratio as measured with TeloView (3D Signatures Inc.). A second study that explains the a/c ratio in relation to the cell cycle phase was done by Vermolen el al. [11]. The authors synchronized and sorted the cells according to their cell cycle phase. The values for *a*/*c* ratio for nuclei in G_0_/G_1_, S, and G_2_ were 1.4 ± 0.1, 1.5 ± 0.2, and 14 ± 2, respectively, showing a significant difference between G_2_ and G_0_/G_1_ or S [11]. In addition, 3D imaging revealed a specific 3D telomeric signature where telomeres did not overlap in normal nuclei and were not misclassified as aggregates when they form a telomeric disk.

In our study, the increase in the a/c ratio can be linked to the acquisition of cytogenetic abnormalities since short telomeres induce aggregates, which consist of fused telomeres or telomeres in very close illegitimate proximity [6,7,11,12,13,14]. This aggregate formation enables both illegitimate recombination and breakage-bridge fusion cycles. Such breakage-bridge fusion cycles result in chromosome breaks and consequently lead to new rearrangements. However, no experiment to address the proliferative status of the cells was performed in the present study.

Among the available methodologies to measure telomere length, the Southern blot of terminal restriction fragments (TRF) methodology requires a large amount of DNA (~3 μg), which limits its applicability in many studies including ours; the qPCR technique estimates the relative ratio of the telomeric repeat amplified sequence to that of a single copy gene, yielding a relative average measurement for the whole cell population. Unlike Southern and qPCR, however, three-dimensional quantitative fluorescence in situ hybridization (3D-Q-FISH) of telomeres combined with TeloView (3D Signatures Inc.) analysis is capable of providing information of single cells and the spatial organization of telomeres in a single nucleus.

In addition, the detection of genome instability can be achieved using a variety of technologies, which range from single-cell approaches to high-throughput multicellular techniques, each one capable of detecting different levels of genomic changes. These measurements are more easily obtainable for cancer cell lines. Measuring genome stability accurately in clinical tumor specimens where material is often limited and substantial cellular heterogeneity exists is considerably more difficult. As a result, only very few methodologies are available and applicable to analysis at a single cell level, karyotyping and single-cell sequencing. Here, we demonstrate that an alternative technology, 3D nuclear telomere profiling, can be used to detect genomic instability in MDS cells. Our results were compared with karyotyping but comparison with single cell sequencing analyses was not part of the present study.

In conclusion, we showed that the 3D nuclear organization of telomeres was significantly altered during the MDS disease course, which may have implications for the acquisition of additional cytogenetic changes. Furthermore, the analysis of 3D telomere architecture appears to be a useful tool for the analysis and possibly early detection of the dynamic changes of the nuclear architecture of telomeres in MDS cells. Larger studies correlating the 3D telomere profiles, cytogenetic evolution and transformation to AML are necessary to better define patients as stable and unstable and to help develop improved therapeutic strategies.

## Figures and Tables

**Table 1:** Baseline characteristics of all MDS patients.

Characteristics	Number	%
**Age at diagnosis**		
**Adults range (19–71)**	11/15	73.33%
**Children (1–18)**	4/15	26.67%
**Gender**		
**Male**	10/15	66.67%
**Female**	5/15	33.33%
**WHO subtype**		
**RCUD**	2/15	13.33%
**RARS**	1/15	6.67%
**RCMD**	6/15	40%
**RAEB-1**	3/15	20%
**RAEB-2**	2/15	13.33%
**MDS-U**	0/15	0
**MDS with isolated del(5q)**	1/15	6.67%
**IPSS-R**		
**Very low**	0/15	0
**Low**	0/15	0
**Intermediate**	13/15	86.68
**High**	1/15	6.66%
**Very high**	1/15	6.66%

MDS with del(5q), myelodysplastic syndromes with deletion of 5q; IPSS-R, revised International Prognostic Scoring System; RAEB, refractory anemia with excess blasts; RARS, refractory anemia with ringed sideroblasts; RCMD, refractory cytopenia with multilineage dysplasia; RCUD, refractory cytopenia with unilineage dysplasia; WHO, World Health Organization.

## Supplementary Materials

The following are available online at https://www.mdpi.com/2073-4409/8/4/304/s1, Table S1: Clinical characteristics of fifteen myelodysplastic syndrome patients with clonal cytogenetic evolution, Table S2: Statistical Analysis of Telomere Parameters in each time point for fifteen myelodysplastic syndrome patients.
