# Supplementary material for: 3D Telomere Structure Analysis to Detect Genomic Instability and Cytogenetic Evolution in Myelodysplastic Syndromes
Source: Cells. 2019 Apr 2;8(4):304. doi: 10.3390/cells8040304 (PMC6526472; doi:10.3390/cells8040304)
Supplement: Supplementary file 1 [file cells-08-00304-s001.pdf]

Table S1. Clinical characteristics of fifteen myelodysplastic syndrome patients with clonal cytogenetic evolution.

MDS Patients	Age	Gender	WHO Subtype	IPSS-R	Cytogenetic	Any Cytogenetic Changes During Disease	Time Lapse Between Samples (Days)	Progression to AML
**P1**								
P1a	10	M	RCMD	INT	46,XY[20]	Yes	P1a vs P1b = 32	Yes
P1b					46,XY[22]		P1b vs P1c = 35	
P1c					46,XY,inv(10)(p11q21.2)[12]/46,XY[13]		P1c vs P1d = 29	
P1d					46, XY,inv(10)(p11q21.2)[10] plus multiple breaks			
**P2**								
P2a	11	F	RAEB-2	High	46,XX[20]	Yes	P2a vs P2b = 28	Yes
P2b					45,XX,-7[18]/46,XX[2]			
**P3**								
P3a	63	M	RAEB-1	INT	46,XY[20]	Yes	P2a vs P2b = 35	No
P3b					46,XY,del(11)(q21)[13]/46,XY[7]			
**P4**								
P4a	55	F	RAEB-1	INT	46,XX[20]	Yes	P4a vs P4b = 36	Yes
P4b					46,XX,i(17)(q10)[18]/46,XX[2]		P4b vs P4c = 31	
P4c					46,XX,i(17)(q10)[26]/46,XX,del(6)(q21q23)[6]/47,XX,+20[2]			
**P5**								
P5a	5	M	RCMD	INT	46,XY[20]	Yes	P5a vs P5b = 40	Yes
P5b					45,XY,-8[13]/46,XY[19]			
**P6**								
P6a	31	F	RAREB-2	INT	46,XX[14]	Yes	P6a vs P6b = 37	No
P6b					47,XX,+15[6]/46,XX[16]			
**P7**								
P7a	82	F	RARS	INT	46,XX[20]	Yes	P7a vs P7b = 48	No
P7b					47,XX,+19[17]/46,XX[10]			
**P8**								
P8a	69	F	5Q- SYND	INT	46,XX,del(5)(q22)[10]/46,XX[12]	Yes	P8a vs P8b = 57	No
P8b					46,XX,del(5)(q21)[4]/47,XX,+8[4]/46,XX[12]			

**P9**<br>P9a<br>P9b	46	M	RAREB-1	Very high	46,XY,der(11)t(1;11)(q21;q23)[23]/46,sl,+21,der(21;21)(q10;q10)[2]/46,XY[5]<br>46,XY,der(11)t(1;11)(q21;q23)[19]/46,sl,+21,der(21;21)(q10;q10)[2]/46,XY[1]	No	P9a vs P9b = 43	No
**P10**<br>P10a<br>P10b	14	M	RCMD	INT	46,XY[24]<br>46,XY,del(11)(q23)[4]/46,XY[16]	Yes	P10a vs P10b = 33	No
**P11**<br>P11a<br>P11b	62	M	RCUD	INT	46,XY[24]<br>46,XY,iso(17q)[10]/46,XY[15]	Yes	P11a vs P11b = 52	No
**P12**<br>P12a<br>P12b	53	M	RCUD	INT	46,XY[20]<br>46,XY[22]	No	P12a vs P12b = 48	No
**P13**<br>P13a<br>P13b	62	M	RCMD	INT	46,XY[25]<br>46,XY[27]	No	P13a vs P13b = 32	No
**P14**<br>P14a<br>P14b	54	M	RCMD	INT	46,XY[23]<br>46,XY[22]	No	P14a vs P14b = 45	No
**P15**<br>P15a<br>P15b	63	M	RCMD	INT	46,XY[20]<br>46,XY[25]	No	P15a vs P15b = 35	No

Table S2. Statistical Analysis of Telomere Parameters in each time point for fifteen myelodysplastic syndrome patients.

MDS Patients	Total Number of Signals		Total Number of Aggregates		Total Intensity		Average Intensity of all Signals		a/c Ratio		Nuclear Volume	
	**Mean**	**Std Dev**	**Mean**	**Std Dev**	**Mean**	**Std Dev**	**Mean**	**Std Dev**	**Mean**	**Std Dev**	**Mean**	**Std Dev**
**P1**												
P1a	51.1875000	2.81	4.31	0.54	898551.560	41473	18013.7526	683	6.91	1.16	305594.219	26409
P1b	56.5937500	2.81	4.58	0.52	832506.500	41473	15813.1412	683	7.51	1.16	322182.324	26409
P1c	35.5937500	2.81	6.03	0.54	563020.875	41473	15196.8115	683	8.20	1.12	349986.313	26409
P1d	40.2647059	2.73	6.78	0.54	427882.794	40234	10741.7303	662	10.13	1.16	455736.594	25621
p-values P1a vs P1b	0.17		0.33		0.26		**0.02**		0.70		0.65	
P1a vs P1c	**0.0009**		**0.05**		**<0.001**		**0.0042**		0.42		0.23	
P1a vs P1d	**0.0062**		**0.02**		**<0.001**		**<0.001**		**0.05**		**<0.001**	
P1b vs P1c	**<0.001**		**0.0017**		**<0.001**		0.52		0.11		0.45	
P1b vs P1d	**<0.001**		**0.0044**		**<0.001**		**<0.001**		0.67		**0.0004**	
P1c vs P1d	0.49		0.71		**0.02**		**<0.001**		0.23		**0.0052**	
**P2**												
P2a	36.4857143	2.9	1.96	0.43	454854.429	33649	12927.1573	684	11.91	0.80	412200.333	50133
P2b	21.4242424	3.0	4.17	0.42	180441.394	34654	8729.3935	705	16.02	0.82	427931.000	51629
p-value P2a vs P2b	**<0.001**		**<0.001**		**<0.001**		**<0.001**		0.30		0.82	
**P3**												
P3a	45.5666667	2.20	5.85	0.59	675100.281	39346	14580.8489	717	4.02	0.71	304639.906	29691
P3b	39.1333333	2.20	5.96	0.56	635317.971	37622	14181.4843	750	6.22	0.68	405023.371	28390
p-value P3a vs P3b	**0.04**		0.89		0.46		0.70		**0.02**		0.01	
**P4**												
P4a	49.8529412	2.3	3.0	0.45	722683.882	36984	15469.6291	727	5.02	0.68	201680.469	20344
P4b	35.0909091	2.2	4.0	0.44	527161.781	35880	14982.7771	705	6.02	0.68	220601.147	19737
P4c	31.0909091	2.3	5.7	0.45	383203.121	36420	12155.3181	716	11.66	0.67	377879.061	20033
p-value P4a vs P4b	**<0.001**		0.14		**0.0003**		0.63		0.29		0.5	
P4a vs P4c	**<0.001**		**<0.001**		**<0.001**		**0.0016**		**<0.001**		**<0.001**	
P4b vs P4c	0.23		**0.0059**		**0.0067**		**0.0060**		**<0.001**		**<0.001**	
**P5**												
P5a	48.1764706	2.36	5.52	0.52	844860.794	40370	17800.0734	668	5.53	0.63	216063.588	18646
P5b	47.8529412	2.36	6.2	0.52	672117.206	40370	14429.5174	668	6.43	0.63	228006.618	18646

p-value P5a vs P5b	0.92		0.36		**0.0035**		**0.0007**		0.32		0.65	
**P6**												
P6a	49.7575758	2.82	5.33	0.59	532059.636	31435	15255.3839	603	16.59	1.66	302437.517	27605
P6b	42.8275862	2.65	5.72	0.55	626876.966	29468	11054.4545	565	23.78	1.66	406541.394	25878
p-value P6a vs P6b	0.07		0.63		**0.03**		**<0.001**		**0.0025**		**0.0078**	
**P7**												
P7a	53.00000000	2.25	5.19	0.53	698621.686	38355	13584.0235	664	6.96	0.70	333984.333	35095
P7b	42.75000000	2.22	7.37	0.52	542221.444	37818	12633.2825	655	9.01	0.69	563562.257	34604
p-value P7a vs P7b	**0.0019**		**0.005**		**0.005**		0.31		**0.04**		**<0.001**	
**P8**												
P8a	58.3823529	3.17	6.68	0.61	628986.156	33212	10634.3926	552	8.55	0.91	348036.176	30183
P8b	53.3823529	3.08	7.14	0.59	568327.912	32220	12037.1853	535	12.65	0.88	407542.125	29282
p-value P7a vs P7b	0.30		0.59		**0.05**		0.07		**0.0019**		0.16	
**P9**												
P9a	39.0333333	2.20	4.56	0.46	370354.900	23257	9599.7528	440	13.98	1.66	777245.80	91403
P9b	43.7000000	2.20	5.13	0.46	419707.100	23257	9991.3067	440	11.61	1.66	755286.20	91403
p-value P9a vs P9b	0.18		0.38		0.17		0.52		0.31		0.86	
**P10**												
P10a	45.0882353	2.30	4.70	0.46	502292.206	24293	11398.7573	487	3.77	0.66	179679.970	23930
P10b	39.0000000	2.26	4.57	0.46	424684.235	24293	6292.5889	479	9.28	0.60	513273.872	22595
p-value P10a vs P10b	0.06		0.75		**0.02**		**<0.001**		**<0.001**		**<0.001**	
**P11**												
P11a	49.5468647	2.26	4.72	0.42	502292.206	24293	11166.3764	432	4.03	0.66	356688.559	23575
P11b	38.4242424	2.26	4.50	0.47	237227.091	23933	8192.7839	405	11.48	0.65	523183.970	23575
p-value P11a vs P11b	0.04		0.84		**<0.001**		**<0.001**		**<0.001**		**<0.001**	
**P12**												
P12a	40.2666667	2.20	4.53	0.46	438558.233	23257	11216.7550	440	11.71	1.66	866292.37	91403
P12b	37.9333333	2.20	4.53	0.46	405378.000	23257	10627.4650	440	10.33	1.66	795224.20	91403
p-value P12a vs P12b	0.45		0.91		0.53		0.34		0.56		0.58	
**P13**												
P13a	42.5333333	2.20	4.86	0.46	407489.600	23257	9722.1153	440	6.11	0.58	883816.07	91403
P13b	37.4333333	2.20	4.56	0.46	349065.267	23257	9403.6284	440	6.31	0.54	882490.60	91403
p-value P13a vs P13b	0.1034		0.64		0.34		0.60		0.83		0.49	
**P14**												

P14a	49.2812500	2.8	5.00	0.46	419574.267	23257	9557.1312	440	19.63	1.66	1130028.40	91403
P14b	45.5714286	2.7	4.56	0.46	369984.767	23257	9558.1164	440	21.50	1.66	1115074.40	91403
p-value P14a vs P14b	0.34		0.50		0.60		0.99		0.42		0.90	
**P15**												
P15a	32.7666667	2.20	3.40	0.46	285431.167	23257	9158.3090	440	18.89	1.66	960903.63	91403
P15b	34.2000000	2.20	3.50	0.46	281497.367	23257	8318.3796	440	17.77	1.66	1082359.40	91403
p-value P15a vs P15b	0.64		0.87		0.99		0.17		0.63		0.34	
